# A unified analytic solution approach to static bending and free vibration problems of rectangular thin plates

**DOI:** 10.1038/srep17054

**Published:** 2015-11-26

**Authors:** Rui Li, Pengcheng Wang, Yu Tian, Bo Wang, Gang Li

**Affiliations:** 1 State Key Laboratory of Structural Analysis for Industrial Equipment, Department of Engineering Mechanics, Dalian University of Technology, Dalian 116024, China; 2 State Key Laboratory of Digital Manufacturing Equipment & Technology, Huazhong University of Science and Technology, Wuhan 430074, China

## Abstract

A unified analytic solution approach to both static bending and free vibration problems of rectangular thin plates is demonstrated in this paper, with focus on the application to corner-supported plates. The solution procedure is based on a novel symplectic superposition method, which transforms the problems into the Hamiltonian system and yields accurate enough results via step-by-step rigorous derivation. The main advantage of the developed approach is its wide applicability since no trial solutions are needed in the analysis, which is completely different from the other methods. Numerical examples for both static bending and free vibration plates are presented to validate the developed analytic solutions and to offer new numerical results. The approach is expected to serve as a benchmark analytic approach due to its effectiveness and accuracy.

Static bending and free vibration problems of thin plates are two types of fundamental issues in mechanical and civil engineering as well as in applied mathematics, with extensive applications such as floor slabs for buildings, bridge decks, and flat panels for aircrafts. In view of their importance, the problems have received considerable attention. Since the governing equations as well as boundary conditions for thin plates have been established long ago, the main focus has been on the solutions, which has brought in a variety of solution methods for various plates. Most of these methods are approximate/numerical ones such as the finite difference method^1^,^2^, the finite strip method^3^,^4^, the finite element method (FEM)^5^,^6^, the boundary element method^7^,^8^, the differential quadrature method^9^,^10^, the discrete singular convolution method^11^,^12^,^13^,^14^, the meshless method^15^,^16^,^17^, the collocation method^18^,^19^,^20^, the Illyushin approximation method^21^,^22^, the Rayleigh-Ritz method and Galerkin method^23^.

In comparison with the prosperity of approximate/numerical methods, analytic methods are scarce for both static bending and free vibration problems of rectangular thin plates. The reason is that the governing partial differential equation for the problems is very difficult to solve analytically except the cases of plates with two opposite edges simply supported, which have the classical Lévy-type semi-inverse solutions. For the plates without two opposite edges simply supported, there exist several representative analytic methods such as the semi-inverse superposition method^24^,^25^, series method^23^, integral transform method^26^, and symplectic elasticity method^27^,^28^,^29^,^30^,^31^,^32^.

It should be noted that many of previous analytic methods are only suitable for one type of static bending and free vibration problems. In this paper, a unified analytic solution approach to static bending and free vibration problems of rectangular thin plates is developed. The approach is implemented in the symplectic space within the framework of the Hamiltonian system. Superposition of two fundamental problems, which are solved analytically, is applied. Therefore, it is referred to as the symplectic superposition approach^33^. It was first proposed to solve the static bending problems^34^,^35^, and was successfully extended to free vibration problems recently^36^. We thus find a way to analytically solve both static bending and free vibration problems in a unified procedure. When the static bending solutions are obtain by the current approach, the free vibration solutions can be readily obtained without extra methodological effort.

To provide new benchmark solutions, we focus on the rectangular thin plates with four corners point-supported, which could rest on an elastic foundation. The investigations on such problems are less common than those on the plates with combinations of free, clamped, and simply supported boundary conditions. Several related references are reviewed here, which provide the solutions by numerical results for validation of our approach. Rajaiah & Rao^37^ used the collocation method with equidistant points along the plate edge to present a series solution to the problem of laterally loaded square plates simply supported at discrete points around its periphery. Lim *et al.*^29^ developed the analytic solutions for bending of a uniformly loaded rectangular thin plate supported only at its four corners, where the symplectic elasticity method was employed and the free boundaries with corner supports were dealt with using the variational principle. Abrate^38^ presented a general approach based on the Rayleigh-Ritz method and the Lagrange multiplier technique to study the free vibrations of point-supported rectangular composite plates. Cheung & Zhou^39^ proposed a new set of admissible functions which were composed of static beam functions to give numerical solutions for the free vibrations of rectangular composite plates with point-supports. It was then further improved to obtain optimal convergence^40^. In an important technical report by Leissa^23^, many conventional solution methodologies for free vibration of plates were introduced, and comprehensive numerical results for the frequencies and mode shapes were presented, including those of corner-supported plates.

In this paper, accurate analytic results for both the static bending and free vibration solutions, validated by the FEM and other solution methods (if any), are tabulated or plotted to serve as the benchmarks for validation and error analysis of various new methods developed in future.

## Hamiltonian system-based governing equations for static bending and free vibration problems of a thin plate

The Hamiltonian variational principle for static bending and free vibration problems of a thin plate on an elastic foundation is in the form^36^,^41^


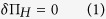


where the mixed energy functional Π_*H*_ is


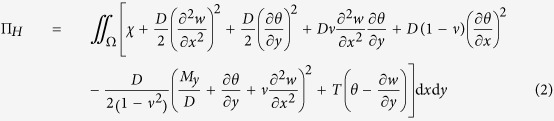


Herein Ω denotes the plate domain; *x* and *y* are the Cartesian coordinates; *w* is the plate’s transverse deflection for static bending problems and is mode shape function for free vibration; *M*_*y*_ is the bending moment; *ν* is the Poisson’s ratio; *D* is the flexural rigidity; *θ* and *T* will be interpreted after equation (4). *χ* equals *Kw*^2^/2 − *qw* for static bending problems and K^*^w^2^/2 for free vibration, where *K* is the Winkler-type foundation modulus; *q* is the distributed transverse load; K^*^ = *K* − *ρhω*^2^, in which *ρ* is the plate mass density, *h* is the plate thickness, and *ω* is the circular frequency. The variations with respect to the independent *w*, *θ*, *T*, and *M*_*y*_, respectively, lead to a matrix equation


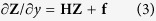


for static bending problems and


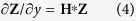


for free vibration, where **Z** = [*w*, *θ*, *T*, *M*_*y*_]^T^, **f** = [0, 0, *q*, 0]^T^, 
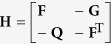
, 
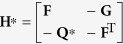
, 
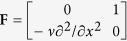
, 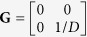, 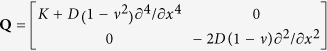, 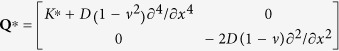. **H** and H^*^ are both the Hamiltonian operator matrices, which satisfy **H**^T^ = **JHJ** and H^*T^ = JH^*^J, respectively. 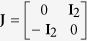 is the symplectic matrix where **I**_2_ is the 2 × 2 unit matrix. One could find from equations (3) and (4) that *θ* = ∂*w*/∂*y*, and *T* = −*V*_*y*_, where *V*_*y*_ is the equivalent shear force^24^. Equations (3) and (4) are the Hamiltonian system-based governing matrix equations for static bending problems and free vibration of a thin plate, respectively.

It is interesting to note that the two governing equations are similar in form; only equation (4) is homogeneous while equation (3) is inhomogeneous. Accordingly, as will be shown in the following, the solution approaches to these two problems are also similar, only different in solving the final simultaneous algebraic equations because one group is homogeneous while the other one is inhomogeneous. We will start with the solution of the inhomogeneous equation (3) and then reduce to the homogeneous case based on the unified analytic approach.

## Symplectic analytic solutions for fundamental problems

### Fundamental problem 1

**Figure 1:**
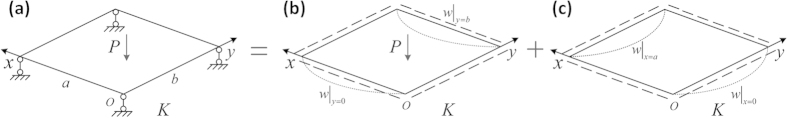
Symplectic superposition for static bending problem of a rectangular thin foundation plate with four corners point-supported.

To solve a rectangular thin foundation plate as shown in Fig. 1a, the foundation plate with two opposite edges simply supported and with given deflections distributed along the other two simply supported edges is regarded as the fundamental problem (Fig. 1b). Our goal is to construct the fundamental solutions for superposition. Without loss of generality, the static bending problem of such a rectangular thin plate subjected to a concentrated load *P* is considered. In the Cartesian coordinate system (*x*, *y*), *x* ∈ [0, *a*] and *y* ∈ [0, *b*]. (*x*_0_, *y*_0_) is the coordinate of load position.

The plate is simply supported along the edges *x* = 0 and *x* = *a*. The bending moment *M*_*y*_ vanishes along the edges *y* = 0 and *y* = *b* but the deflections represented by 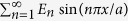 and 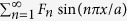 are distributed along the two edges, respectively.

An eigenvalue problem **HX**(*x*) = *μ***X**(*x*) in combination with *dY*(*y*)/*dy* = *μY*(*y*) determines a variable separation solution, **Z** = **X**(*x*) *Y*(*y*), for ∂**Z**/∂*y* = **HZ**. Herein **X**(*x*) = [*w*(*x*), *θ*(*x*), *T*(*x*), *M*_*y*_(*x*)]^T^ depends only on *x*, and *Y*(*y*) only on *y*. The eigenvalues *μ* and corresponding eigenvectors **X**(*x*) satisfying the boundary conditions 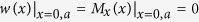 are


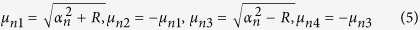


and


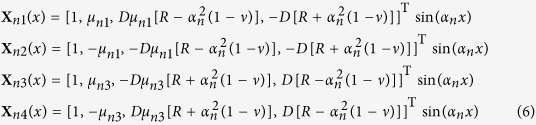


for *n* = 1, 2, 3, ···, where *α*_*n*_ = *nπ*/*a* and 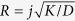, *j* is the imaginary unit.

The solution of equation (3) is


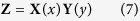


where


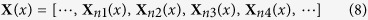



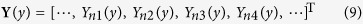


**Y** is determined by


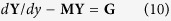


by substituting equation (7) into equation (3) and using **HX** = **XM** and **f** = **XG**, where **M** = diag(···, *μ*_*n*1_, *μ*_*n*2_, *μ*_*n*3_, *μ*_*n*4_, ···), and **G** = [···, *g*_*n*1_, *g*_*n*2_, *g*_*n*3_, *g*_*n*4_, ···]^T^ is the expansion coefficients of **f**.

For the concentrated load *P*, 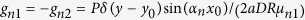, and 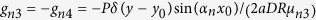, where *δ*(*y* − *y*_0_) is the Dirac delta function. Thus we have, from equation (10),


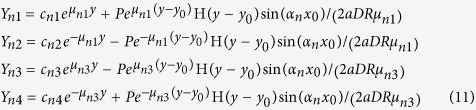


where H(*y* − *y*_0_) is the Heaviside theta function. The constants *c*_*n*1_–*c*_*n*4_ are determined by substituting equations (6) and (11) into equations (8) and (9) then equation (7), and using the boundary conditions at *y* = 0 and *y* = *b*:


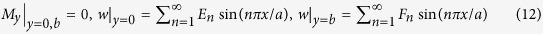


In this way we obtain the analytic solution of the first fundamental problem:


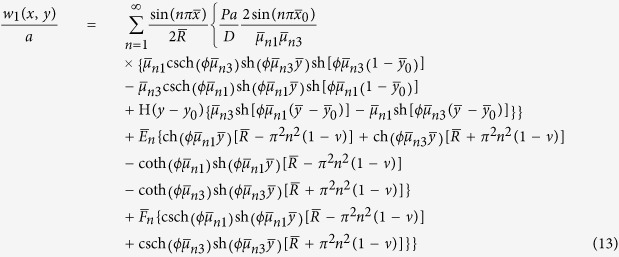


where 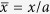, 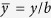, 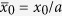, 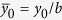, *ϕ* = *b*/*a*, 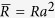, 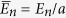, 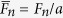, 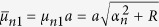, and 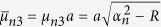.

### Fundamental problem 2

When *P* = 0, the solution of the first fundamental problem reduces to that of the second fundamental problem, i.e., an unloaded rectangular thin foundation plate with the same boundary conditions as in the first fundamental problem (Fig. 1c). By interchanging *x* and *y* as well as *a* and *b*, and replacing *E*_*n*_ and *F*_*n*_ with *G*_*n*_ and *H*_*n*_, respectively, we have the solution of the plate simply supported along the edges *y* = 0 and *y* = *b*, with the bending moment *M*_*x*_ vanishing along the edges *x* = 0 and *x* = *a* but the deflections represented by 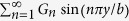 and 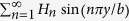 distributed along the two edges, respectively. This solution is


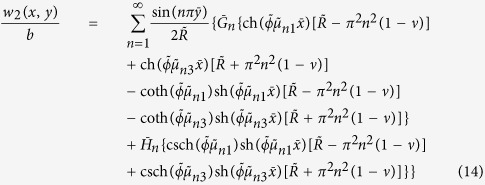


where 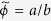, 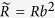, 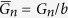, 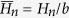, 
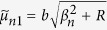
, and 
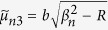
, in which *β*_*n*_ = *nπ*/*b*.

Setting *P* = 0 and using K^*^ instead of *K* in equations (13) and (14), the corresponding mode shape solutions for free vibration problems are readily obtained, i.e.,


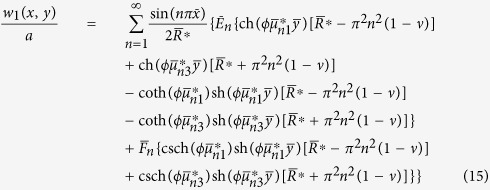


and


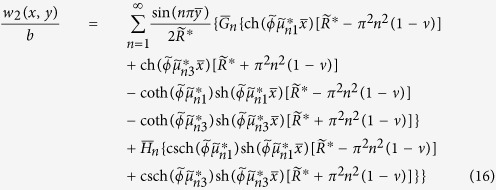


where the quantities with an asterisk are those with K^*^ instead of *K*, i.e., 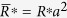, 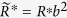, 
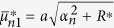
, 
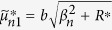
, 
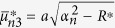
, and 
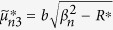
, in which 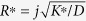.

## Symplectic superposition for analytic solutions of static bending and free vibration problems of corner-supported plates

The analytic solutions of the two fundamental problems have been obtained in section 3. The original problem’s solution is given by


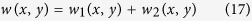


where the constants *E*_*m*_, *F*_*m*_, *G*_*n*_, and *H*_*n*_ (*m*, *n* = 1, 2, 3, ···) are to be determined by imposing the original boundary conditions along each edge. Here the subscripts “*m*” and “*n*” are used to differentiate between the constants of the two fundamental problems.

### Static bending problems

To satisfy the conditions that the equivalent shear force *V*_*y*_ must be zero along the free edges *y* = 0 and *y* = *b* and *V*_*x*_ be zero along the free edges *x* = 0 and *x* = *a*, we obtain a set of 2*M* + 2*N* simultaneous algebraic equations to determine *E*_*m*_, *F*_*m*_, *G*_*n*_, and *H*_*n*_ after truncating the infinite series at *m* = *M* terms and *n* = *N* terms, respectively. These equations are


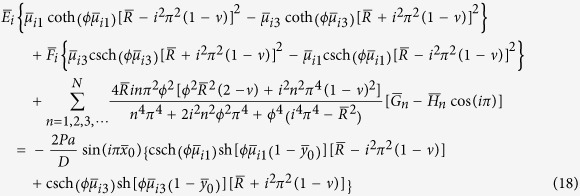


and


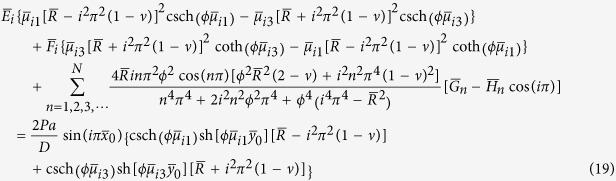


for *i* = 1, 2, 3, ···, *M*, and


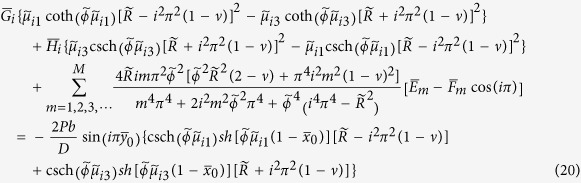


and


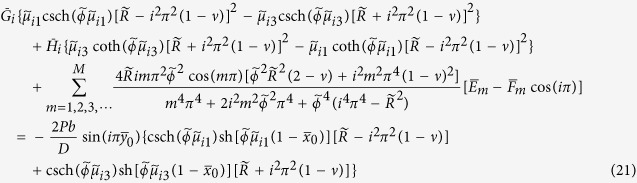


for *i* = 1, 2, 3, ···, *N*, where 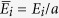, 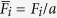, 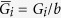, 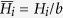, 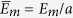, 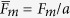, 
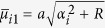
, 
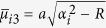
, 
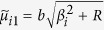
, and 
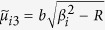
, in which *α*_*i*_ = *iπ*/*a* and *β*_*i*_ = *iπ*/*b*. For simplification, we take *M* = *N* in calculation.

### Free vibration problems

Based on the solutions we have obtained for static bending problems, it is easy to solve free vibration problems by setting *P* = 0 and using K^*^ instead of *K* throughout the solution procedure. The updated equations of equations (18)–(21), 56, 57, 58 become homogeneous, and the frequency parameters are within the coefficient matrix. This is different from static bending problems where the inhomogeneous equations are directly solved with a unique solution. The determinant of the coefficient matrix is set to be zero to yield the frequency equation. Substituting one of the frequency solutions back into the homogeneous equations, a nonzero solution comprising a set of *E*_*m*_, *F*_*m*_, *G*_*n*_, and *H*_*n*_ is obtained. Substituting them into equation (17) gives the corresponding mode shape function.

It should be noted that proper manipulation of the above simultaneous algebraic equations will lead to analytic solutions of more static bending and free vibration problems of point-supported plates with simply supported edges. For example, setting *F*_*m*_ = 0 and eliminating equation (19) to solve for *E*_*m*_, *G*_*n*_, and *H*_*n*_, we obtain the analytic solution of the plate with two adjacent corners point-supported and their opposite edge simply supported by using equation (17). Setting *F*_*m*_ = 0 and *H*_*n*_ = 0, and eliminating equations (19) and (21) to solve for *E*_*m*_ and *G*_*n*_, we obtain the analytic solution of the plate with a corner point-supported and its two opposite edges simply supported.

## Numerical examples and Discussion

A square thin plate with four corners point-supported under a central concentrated load is solved as the first representative static bending problem. Poisson’s ratio *ν* = 0.3 is taken throughout the study. Nondimensional deflections and bending moments at different locations are tabulated in Table 1. We first compare the analytic results with those available in the literature^37^, where the collocation method was applied to obtain the deflection distribution along the central line of the plate. Very good agreement is observed. The small errors are probably due to the approximation of the collocation method itself. Noting that there are only six data available in ref. 37 for the current problem, we perform the refined finite element analysis by the FEM software package ABAQUS^42^ to further validate our solutions. The 4-node shell element S4R and 200 × 200 uniform mesh (i.e., with the element size of 1/200 of the plate width) are adopted. Excellent agreement is observed between all the current solutions and those by FEM. It should be noted in Table 1 that the bending moment at the concentrated load position does not converge due to singularity^24^.

**Figure 2:**
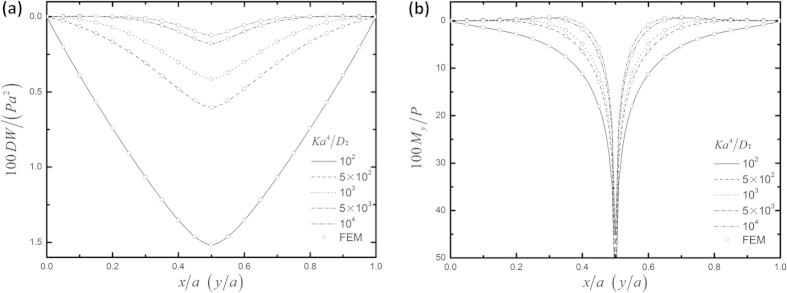
Distribution of (**a**) nondimensional deflections and (**b**) nondimensional bending moments along the diagonal of a square thin foundation plate with four corners point-supported, with *Ka*^4^/*D* = 10^2^, 5 × 10^2^, 10^3^, 5 × 10^3^, and 10^4^, respectively.

The second example is a square thin foundation plate with four corners point-supported under a central concentrated load, with the nondimensional foundation modulus *Ka*^4^/*D* = 10^2^. The analytic results are tabulated in Table 2 by comparison with those by FEM only since we did not find any such solutions in the literature. Excellent agreement is also observed for all the results. It is convenient to use the above analytic solutions to investigate the effect of *K* on the plate solutions. As shown in Fig. 2, nondimensional deflections (Fig. 2a) and bending moments (Fig. 2b) along the diagonal of a square thin foundation plate are plotted for *Ka*^4^/*D* = 10^2^, 5 × 10^2^, 10^3^, 5 × 10^3^, and 10^4^. Again, excellent agreement with FEM is observed.

**Figure 3:**
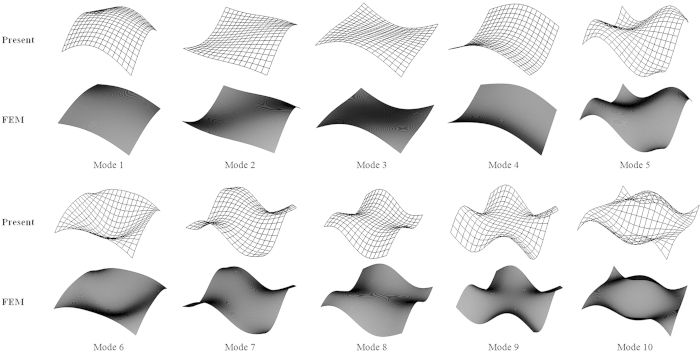
First ten mode shapes of a square thin plate with four corners point-supported.

To illustrate the applicability of the method to free vibration, we calculate the first ten frequency parameters of corner-supported rectangular foundation plates with the aspect ratios ranging from 1 to 5, as shown in Table 3. The validity and accuracy of the current method are confirmed in view of the excellent agreement with the literature^23^,^38^,^39^,^40^ and, especially, with FEM. The first ten mode shapes of a square thin plate are shown in Fig. 3, which have also been validated by FEM.

**Figure 4:**
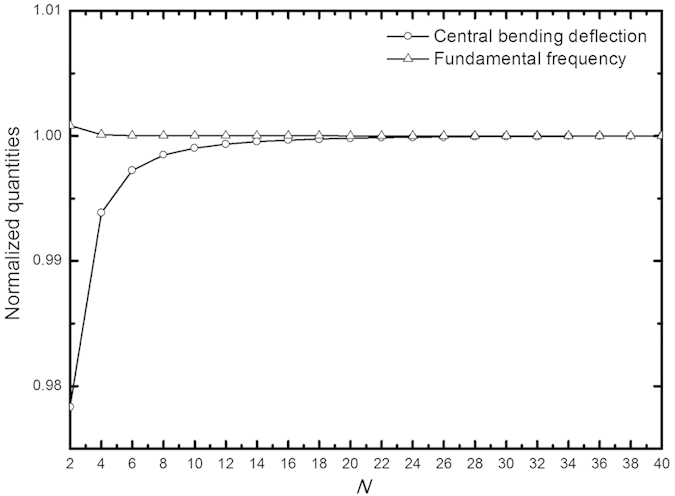
Convergence of the normalized bending and free vibration solutions of a square thin plate with four corners point-supported.

An important issue concerned in solving the above problems is the convergence of the solutions. To examine it, we investigate a square corner-supported plate. Figure 4 illustrates the normalized central bending deflection and fundamental frequency versus the series terms adopted in calculation. It is seen that both the bending and free vibration solutions converge rapidly since only dozens of terms are enough to furnish satisfactory convergence. Actually rapid convergence is found for most solutions. The maximum number of series terms is taken to be 100 to achieve the convergence of all current numerical results to the last digit of five significant figures.

## Conclusions

A unified analytic approach is developed in this paper to solve static bending and free vibration problems of rectangular thin plates. The approach combines the Hamiltonian system-based symplectic method and the superposition, and has the exceptional advantage that no trial solutions are needed in the analysis. Therefore, it provides a rational way to yield the analytic solutions. The procedures for the two kinds of problems are similar except in solving the equations in terms of undetermined constants. For static bending problems, the equations are inhomogeneous and a unique solution could be directly obtained, while for free vibration problems, the equations are homogeneous and the condition of having nonzero solutions is imposed to give the frequencies before solving for nonzero solutions, for which the determinant of the coefficient matrix is set to be zero to yield the frequency equation. The resultant key quantities for static bending problems are the transverse deflection and its derivatives while those for free vibration problems are the frequencies and associated mode shapes. It is seen that the proposed approach is very effective and accurate for rectangular thin plate problems. It is expected to serve as a benchmark analytic approach to similar problems.

## Additional Information

**How to cite this article**: Li, R. *et al.* A unified analytic solution approach to static bending and free vibration problems of rectangular thin plates. *Sci. Rep.* **5**, 17054; doi: 10.1038/srep17054 (2015).

## Figures and Tables

**Table 1:** Bending solutions of a square thin plate with four corners point-supported, having a concentrated load at the plate center.

*y*	*x*	*DW*/(*Pa*^2^)			*M*_*y*_/*P*	
		Present	FEM	Rajaiah and Rao^37^^*^	Present	FEM
0	0	0	0	—	0	0.0035126
	0.1*a*	0.0071903	0.0071900	—	0	0.0014687
	0.2*a*	0.013576	0.013575	—	0	0.0012146
	0.3*a*	0.018595	0.018595	—	0	0.0010491
	0.4*a*	0.021807	0.021806	—	0	0.00092456
	0.5*a*	0.022913	0.022912	—	0	0.00087437
0.1*a*	0	0.0071903	0.0071900	—	0.074548	0.074075
	0.1*a*	0.013084	0.013083	—	0.058633	0.058633
	0.2*a*	0.018535	0.018535	—	0.050263	0.050263
	0.3*a*	0.022945	0.022944	—	0.044869	0.044870
	0.4*a*	0.025822	0.025821	—	0.040974	0.040977
	0.5*a*	0.026823	0.026822	—	0.039403	0.039408
0.2*a*	0	0.013576	0.013575	—	0.12512	0.12472
	0.1*a*	0.018535	0.018535	—	0.11065	0.11065
	0.2*a*	0.023308	0.023307	—	0.10110	0.10110
	0.3*a*	0.027307	0.027306	—	0.094551	0.094552
	0.4*a*	0.029992	0.029991	—	0.089212	0.089211
	0.5*a*	0.030944	0.030942	—	0.086676	0.086680
0.3*a*	0	0.018595	0.018595	—	0.16555	0.16519
	0.1*a*	0.022945	0.022944	—	0.15402	0.15402
	0.2*a*	0.027307	0.027306	—	0.14871	0.14871
	0.3*a*	0.031118	0.031117	—	0.14774	0.14775
	0.4*a*	0.033793	0.033792	—	0.14619	0.14619
	0.5*a*	0.034774	0.034772	—	0.14356	0.14356
0.4*a*	0	0.021807	0.021806	—	0.19307	0.19275
	0.1*a*	0.025822	0.025821	—	0.18468	0.18467
	0.2*a*	0.029992	0.029991	—	0.18652	0.18652
	0.3*a*	0.033793	0.033792	—	0.19998	0.19999
	0.4*a*	0.036633	0.036631	—	0.22054	0.22056
	0.5*a*	0.037762	0.037760	—	0.22516	0.22519
0.5*a*	0	0.022913	0.022912	0.022908	0.20299	0.20269
	0.1*a*	0.026823	0.026822	0.026818	0.19605	0.19604
	0.2*a*	0.030944	0.030942	0.030938	0.20189	0.20188
	0.3*a*	0.034774	0.034772	0.034765	0.22652	0.22651
	0.4*a*	0.037762	0.037760	0.037755	0.28770	0.28769
	0.5*a*	0.039142	0.039140	0.039135	—	—

^*^The results are divided by 40 to yield the same form of nondimensional solutions as that of the present ones.

**Table 2:** Bending solutions of a square thin foundation plate with four corners point-supported, having a concentrated load at the plate center (*Ka* ^4^/*D* = 10^2^).

*y*	*x*	*DW*/(*Pa*^2^)		*M_y_* /*P*	
		Present	FEM	Present	FEM
0	0	0	0	0	0.00082337
	0.1*a*	0.0020321	0.0020321	0	0.00033223
	0.2*a*	0.0038683	0.0038684	0	0.00023269
	0.3*a*	0.0053487	0.0053486	0	0.00012820
	0.4*a*	0.0063190	0.0063189	0	0.000029644
	0.5*a*	0.0066581	0.0066580	0	0.000013229
0.1*a*	0	0.0020321	0.0020321	0.017944	0.017841
	0.1*a*	0.0039016	0.0039017	0.014125	0.014129
	0.2*a*	0.0056615	0.0056616	0.011396	0.011409
	0.3*a*	0.0071312	0.0071312	0.0086268	0.0086308
	0.4*a*	0.0081213	0.0081213	0.0059495	0.0059198
	0.5*a*	0.0084728	0.0084727	0.0046849	0.0046921
0.2*a*	0	0.0038683	0.0038684	0.032383	0.032313
	0.1*a*	0.0056615	0.0056616	0.029734	0.029729
	0.2*a*	0.0074229	0.0074230	0.027893	0.027900
	0.3*a*	0.0089650	0.0089652	0.025733	0.025749
	0.4*a*	0.010051	0.010051	0.022548	0.022592
	0.5*a*	0.010448	0.010448	0.020717	0.020715
0.3*a*	0	0.0053487	0.0053486	0.046639	0.046603
	0.1*a*	0.0071312	0.0071312	0.046421	0.046426
	0.2*a*	0.0089650	0.0089652	0.049193	0.049191
	0.3*a*	0.010669	0.010669	0.053288	0.053298
	0.4*a*	0.011953	0.011954	0.054428	0.054422
	0.5*a*	0.012449	0.012449	0.052606	0.052619
0.4*a*	0	0.0063190	0.0063189	0.058024	0.058017
	0.1*a*	0.0081213	0.0081213	0.060625	0.060639
	0.2*a*	0.010051	0.010051	0.070501	0.070490
	0.3*a*	0.011953	0.011954	0.089133	0.089142
	0.4*a*	0.013530	0.013530	0.11250	0.11251
	0.5*a*	0.014216	0.014216	0.11798	0.11800
0.5*a*	0	0.0066581	0.0066580	0.062480	0.062484
	0.1*a*	0.0084728	0.0084727	0.066438	0.066436
	0.2*a*	0.010448	0.010448	0.080205	0.080215
	0.3*a*	0.012449	0.012449	0.11002	0.11002
	0.4*a*	0.014216	0.014216	0.17396	0.17399
	0.5*a*	0.015167	0.015168	—	—

**Table 3:** Frequency parameters, 
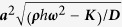
 , of some rectangular thin foundation plates with four corners point-supported.

*b*/*a*	References	Mode									
		1st	2nd	3rd	4th	5th	6th	7th	8th	9th	10th
1	Present	7.1109	15.770	15.770	19.596	38.432	44.370	50.377	50.377	69.265	80.361
	FEM	7.1112	15.769	15.769	19.598	38.440	44.371	50.397	50.397	69.286	80.393
	Leissa^23^,^a^	7.117	15.73	—	19.13	38.42	43.55	—	—	—	—
	Leissa^23^,^b^	7.46	16.80	16.80	19.60	41.5	48.3	51.6	—	—	—
	Leissa^23^,^c^	7.12	15.77	15.77	19.60	38.44	44.4	50.3	—	—	—
	Abrate^38^	7.1109	15.7703	15.7703	19.5961	—	—	—	—	—	—
	Cheung and Zhou^39^	7.136	15.800	15.805	19.710	38.653	44.430	—	—	—	—
	Zhou^40^	7.132	15.797	15.797	19.629	38.562	44.382	—	—	—	—
1.5	Present	3.9669	9.5722	11.475	14.974	23.438	25.651	31.146	39.814	48.999	50.185
	FEM	3.9670	9.5710	11.475	14.975	23.439	25.655	31.148	39.825	49.018	50.190
	Leissa^23^,^a^,*	3.9676	—	—	—	—	—	—	—	—	—
	Leissa^23^,^b^,*	4.0933	10.124	12.329	15.467	24.889	25.644	33.511	—	—	—
	Leissa^23^,^c^,*	3.9644	9.5689	11.476	14.973	23.422	25.644	31.16	—	—	—
2	Present	2.3227	6.8737	8.2057	12.969	15.949	17.811	24.773	27.922	31.280	37.242
	FEM	2.3227	6.8729	8.2060	12.969	15.948	17.812	24.778	27.925	31.284	37.247
	Leissa^23^,^a^,*	2.3233	—	—	—	—	—	—	—	—	—
	Leissa^23^,^b^,*	2.365	7.2575	8.675	14.5	16.775	18.25	—	—	—	—
	Leissa^23^,^c^,*	2.3225	6.875	8.2075	13	15.95	17.825	—	—	—	—
2.5	Present	1.5014	5.3778	5.7287	10.818	11.980	14.665	19.244	20.502	26.285	28.163
	FEM	1.5014	5.3772	5.7289	10.818	11.980	14.666	19.246	20.502	26.287	28.170
	Leissa^23^,^b^,*	1.5168	5.68	0.5952	16.272	12.56	15.6	—	—	—	—
	Leissa^23^,^c^,*	1.5024	—	—	—	—	—	—	—	—	—
3	Present	1.0458	4.1041	4.4250	8.5353	9.5902	12.586	15.934	16.009	21.395	23.878
	FEM	1.0458	4.1043	4.4245	8.5358	9.5895	12.586	15.935	16.009	21.398	23.879
	Leissa^23^,^a^,*	1.0477	—	—	—	—	—	—	—	—	—
3.5	Present	0.76915	3.0530	3.7631	6.6196	8.0046	10.620	13.093	13.881	17.414	19.238
	FEM	0.76915	3.0531	3.7627	6.6199	8.0039	10.620	13.092	13.881	17.416	19.238
4	Present	0.58907	2.3501	3.2757	5.1914	6.8771	8.7502	11.072	12.149	15.062	16.040
	FEM	0.58907	2.3501	3.2754	5.1916	6.8765	8.7507	11.071	12.149	15.063	16.040
4.5	Present	0.46546	1.8614	2.9013	4.1496	6.0339	7.1689	9.5963	10.462	13.356	13.735
	FEM	0.46546	1.8614	2.9010	4.1497	6.0334	7.1693	9.5957	10.462	13.357	13.735
5	Present	0.37701	1.5094	2.6044	3.3811	5.3787	5.9156	8.4734	8.8920	11.821	12.006
	FEM	0.37701	1.5094	2.6041	3.3812	5.3783	5.9159	8.4728	8.8925	11.822	12.006

^a^From the finite difference method.

^b^From the Rayleigh-Ritz method.

^c^From the series method.

^*^The results are divided by (*b*/*a*)^2^ to yield the same form of nondimensional solutions as that of the present ones.
